# Screening of Primary School Children for Amblyopia and Amblyogenic Factors in Central Cairo, Egypt

**DOI:** 10.1155/2018/8425319

**Published:** 2018-04-22

**Authors:** Mohammad A. Rashad, Khaled M. Abd Elaziz, Samah Mahmoud Fawzy, Ahmed Abdel Meguid Abdel latif, Mahmoud Abdel Meguid Abdel latif

**Affiliations:** ^1^Ophthalmology Department, Faculty of Medicine, Ain Shams University, Cairo, Egypt; ^2^Community Department, Environmental and Occupational Medicine, Faculty of Medicine, Ain Shams University, Cairo, Egypt

## Abstract

**Objective:**

To measure the prevalence of amblyopia and amblyogenic factors among primary school children and to evaluate distance visual acuity (VA) as a screening test to detect amblyopia and define its cutoff value.

**Subjects and Methods:**

A cross-sectional study was conducted on primary school children in two schools in Central Cairo. Children underwent assessment of visual acuity using Landolt broken ring. Comprehensive ophthalmologic examination was performed for amblyopia suspects at the Ophthalmology Department of Ain Shams University Hospitals, including reassessment of best-corrected visual acuity (BCVA) using the same chart.

**Results:**

A total of 352 children were examined. Reduced screening VA (amblyopia suspect) was detected in 47 subjects (13.35%) proved amblyopia after comprehensive examination was 1.98% (7 cases). Refractive errors (REs) were present in all suspected and proved amblyopia cases (100%) but was only present in 11.6% of nonamblyopic students (*P* < 0.05). The prevalence of hyperopia in the whole sample was 3.6%, and was 27.6% in subjects with RE. Thirty percent of hyperopic eyes were amblyopic. The prevalence of myopia was 9.3% of the whole sample and 70% of students with RE. Only 9% of myopic eyes were amblyopic. Mild to moderate amblyopia (VA better than 0.2log MAR) was 42.9%, while severe amblyopia represented 57.1%.

**Conclusion:**

This study emphasizes the importance of school-based eye care system targeting the detection of amblyopia by application of a fast screening distance VA test with a cutoff value of high sensitivity at log MAR 0.539 (Snellen′s VA equivalent 6/18).

## 1. Introduction

Amblyopia is a potentially treatable decrease in BCVA with no structural pathology [1]. It can be unilateral if two or more line difference in best-corrected visual acuity (BCVA) is present between the two eyes or bilateral if BCVA is equal or less than 6/12 in both eyes [2, 3]. The basic mechanisms in amblyopia could be interactive anomaly between eyes or dissociative disorder in one or both eyes. It is either anisometropic, strabismic, or visual deprivation in nature [4, 5].

Early detection of amblyopia is important for effective treatment; however, it can be still treated in older age [6]. Recent evidences on successful treatment of amblyopia in children up to 12 years old and more encouraged screening programs for older children [7, 8]. This helps discovering undiagnosed amblyopes who have been missed in earlier screening programs or those living in countries with poor medical services and no screening programs [7, 8]. Although the global international prevalence of amblyopia is 1.6–3.6% [9, 10], the available data concerning the prevalence of amblyopia and RE (refractive error) in different geographical areas is still inadequate.

According to the action plan released by the WHO Global Initiative for the Elimination of Avoidable Blindness in 2006–2011, uncorrected refractive errors are the second cause of global blindness (18%) and the main cause of visual impairment in children between 5 and 15 years of age. There are at least 13 million of them all over the world, of which more than 90% live in developing countries [8]. WHO announced refractive errors as one of the five conditions of immediate management priority and recommended that services for their detection should be integrated in different levels of eye-care provision with special emphasis on primary and secondary school eye-health programs especially in developing countries [8]. However, their plans met some limitations: inadequate population-based prevalence studies, uncertainty of the best cost effective ways to deliver refraction services in those different settings, and lack of awareness among people and the community about proper detection and preventive measures [8].

Following these recommendations, we constructed our study to measure the prevalence of amblyopia and refractive errors as an amblyogenic factor in primary school children in Central Cairo, Egypt, which is a crowded, viable area deficient in such studies. At older age children as our study population, causes of amblyopia other than refractive errors are unlikely, as by this age strabismus and organic causes of visual deprivation should have been discovered and managed. We aimed at identifying the ideal cutoff visual acuity (VA) value for suspecting amblyopia by simply using distance VA chart (Landolt's broken rings), and hence referral to comprehensive examination. A secondary goal of our study is to test the efficacy of Landolt's broken ring chart as a suitable screening tool being simple, low cost, and readily available test at all Egyptian primary schools.

## 2. Subjects and Methods

Our cross-sectional study was based on multistage sampling. It was carried out in Central Cairo, Egypt. We performed this study in two primary schools, one public and one private school with different socioeconomic standards. Both schools were selected from a list of Central Cairo Educational Directorate, using simple random sampling. Systematic random sampling was also used for children in classes.

The total number of students in both schools was 815 students, 315 in the public school and 500 in the private school. Assuming the prevalence of amblyopia ranges from 1% to 5% [10], with alpha error 5% and power of study 80%, our sample size was calculated to be 378 students. One general ophthalmologist performed the screening and the examination.

The first stage was history taking from children, including name, age, history of wearing glasses, and history of squint or surgery. The second stage was examination using simple pin light for gross media opacity, lid position, and pupillary light reflex. Ocular motility examination and cover uncover test were also performed.

**Figure 1:**
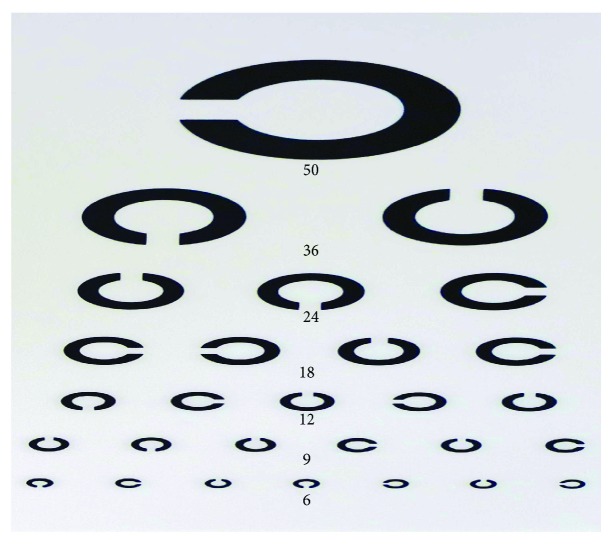
Landolt broken ring (C optotype).

The third stage involved the assessment of monocular uncorrected VA and BCVA with glasses for students who were wearing glasses. Assessment of VA was done at the school premises using Landolt broken ring self-illuminated chart at six meters in a dark room at school (Figure 1). The test was performed monocularly using bracketing technique.

The BCVA of each student was recorded and converted to logarithm minimal angle of resolution (log MAR). Suspected cases were defined as students who had difference in BCVA between both eyes of two lines or more, or those who have BCVA of 6/12 or less in both eyes.

The fourth stage was referring amblyopia suspects to ophthalmic comprehensive examination at Ain Shams University Hospitals. Distant BCVA and pinhole VA were assessed using Landolt broken ring chart. Refraction before and after cycloplegia was performed using both autorefractometer and streak retinoscopy. Cyclopentolate 1% one drop was instilled at 10 minutes interval, repeated for 3 times. Postmydriatic test was performed after one week. Slit lamp and fundus examinations were performed with dilated pupils. Children with reduced BCVA with no organic eye disease are considered proven amblyopia cases to be included in the statistical analysis.

Unilateral amblyopia was defined as ≥2 line difference in BCVA between the two eyes, and bilateral amblyopia as BCVA worse than 6/12 in both eyes. Significant RE was defined as myopia if ≥−0.50 diopter spherical equivalent (D SE), hyperopia if ≥1.0 DSE and astigmatism if ≥1.0 diopter of cylinder (DC).

### 2.1. Statistical Methods

Data was revised for its completeness and consistency. Double data entry on SPSS program version 16 was done. Quantitative data was summarized by mean standard deviation, qualitative data was summarized by frequencies and percentages, and significance was analysed by chi-square test. A “*P* value” of less than 0.05 was considered statistically significant. Sensitivity, specificity, and positive and negative predictive values were also calculated. Distant visual acuity log MAR and log MAR ROC curve analysis were plotted.

## 3. Ethical Considerations

The presented study received the official approval of the Central Cairo Educational Directorate of the screened schools, the two administrators of the selected schools, parents of screened children at school, and the Ethical Committee of the Faculty of Medicine, Ain Shams University. Parents (guardians) of children who had the comprehensive examination provided written informed consents allowing their children to be further examined.

## 4. Results

Our study included initially 378 children between 8 and 12 years of age. Dropouts were 4 children, and absent students were 24. Our actual final sample size was 352 students; 191 public school students (54.3%) and 161private school students (45.7%). The males were 193 (54.8%) and females were 159 students (45.2%). Amblyopia suspects were 47 students (13.4% with 95% CI 9.9–17.3), while7 children were proven amblyopia cases (1.98% with 95% CI 0.8–4.1). Mild to moderate amblyopia (VA better than 0.2 log MAR) was 42.9%, while severe amblyopia represented 57.1%. All proved amblyopia cases were unilateral.

The mean age was 9.5 years among the 345 nonamblyopic children and 9.4 years in the 7 amblyopic cases, with no statistically significant difference. Amblyopia prevalence was higher in the private school and in females. It was also significantly higher in children wearing glasses as shown in Table 1.

The total percentage of RE among all studied students was 13.4%. Refractive errors were present in 100% of suspected and proved amblyopia patients. In nonamblyopic children, only 11.6% (40/345) had RE. The difference was highly significant (*P* < 0.01 chi-square test). The prevalence of hyperopia, myopia, anisometropia, and astigmatism in all students and in cases with RE is illustrated in Table 2.

Amblyopia was more common in hyperopic children 30.7% compared to myopics 9% (*P* value is 0.0867). Amblyopia prevalence in each type of RE is illustrated in Table 3.

**Figure 2:**
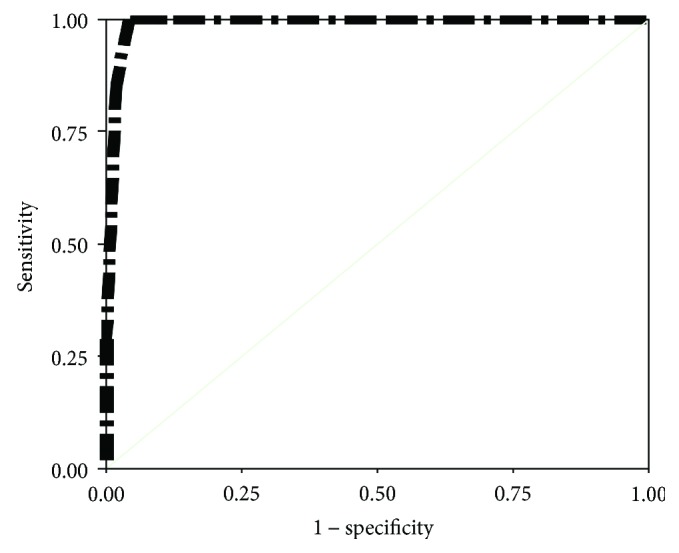
ROC curve sensitivity analysis for VA in detection of amblyopia (all children).

**Figure 3:**
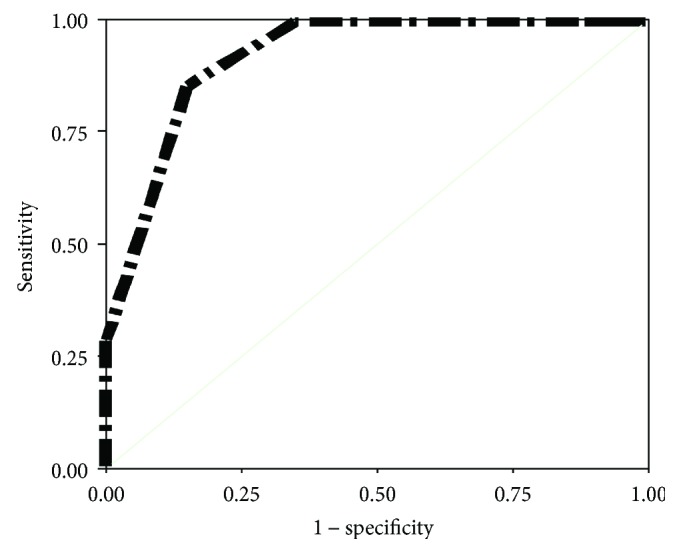
ROC curve sensitivity analysis for VA in detection of amblyopia in suspected subjects.

Validity of our screening test was evaluated by specificity, sensitivity, positive predictive value (PPV), and positive predictive value (NPV) in different cutoff values for detection of suspected and proved cases of amblyopia as shown in Table 4 and Figures (2) and (3). At low cutoff value (0.539 log MAR), sensitivity was 100% at specificity 96% in detection of proved amblyopia. At high cutoff value (0.238 log MAR), sensitivity was also 100% but with 89% specificity in the detection of proved amblyopia.

## 5. Discussion

Vision screening in children is recommended for detection of potentially treatable disorders [8, 11]. In a large population country with moderate resources like Egypt, lack of early eye care services for preschoolers makes the selected age group of our study (8–12 years old); ideal for screening because of their mandatory presence at schools at regular basis, reliable responses, and possible treatment of discovered amblyopia as proven by recent researches [7]. Moreover, in a society like ours, ease of examination with a single, fast, low cost, and readily available test is necessary for successful screening.

In our study, the amblyopia rate in Central Cairo was found to be 1.98%, which is very close to the Menia city in south of Egypt where it was reported to be 1.49% in primary school children [12]. They screened children of narrower age range (7–9 years) and used different screening test, Kay pictures that is not available in Egyptian primary schools [12]. They also reported a higher rate of amblyopia in rural areas (2%) compared to urban areas (1.04%), although they did not state whether this difference was significant or not.

A much lower prevalence of amblyopia was reported in another Egyptian study conducted in Alexandria in north of Egypt where it was only 0.1% in school children 6–12 years of age [13]. Astonishingly, screen failures in their study were only 6% in comparison to 13.4% (CI 9.9–17.3%) in our study [13]. Underestimation of the actual prevalence is suspected in their study as they used a team of technicians of questionable experience, who used Snellen or Tumbling E optotype VA charts for screening [13].

The prevalence of amblyopia in our study is consistent with international results of 1.6–3.6% [8]. It is very comparable to regional developing countries like Saudi Arabia (1.85% in 6–12 years children) [14] and Central China (2.16% in primary school children) with nearly similar prevalence of severe amblyopia to ours (36% and 42%, resp.) [15]. It is also quite similar to developed countries as Sweden where prevalence of deep amblyopia (VA</=0.3) is 2%, reduced to 0.2% with treatment [16].

In our study the prevalence of refractive errors was 13.4% in general, which is lesser than the 22.5% reported in the El-Bayoumy et al. study who screened older school children in Cairo [17] and found that 85.4% of the reported RE was in children ≥ 12 years. We had almost the same prevalence of hyperopia, although more myopia and astigmatism were found in our study (70.2% versus 55.7% and 25.5% versus 17%) [17]. Setting a definite value for every refractive error in our study might have contributed to better discovery of cases as El-Bayoumy et al. [17] did not define values for the included RE nor screened for amblyopia.

Our results were quite similar to Goh et al. in Malaysia [18] who conducted their study by retinoscopy on children aged 7 years. On the other hand, we had more myopia and less hyperopia than Elsahn [13] in north of Egypt (myopia: 70.2% versus 22.0% and hyperopia: 27.6% versus 52%, resp.), which may be due to the slightly older age range of our study children in addition to his questionable screening team [13]. We also had more astigmatism than Elsahn (25.5% of RE versus 10%) as he included cases with pure astigmatism while we included all astigmatic types [13]. We also reported a much higher anisometropia incidence than him [13] (61.7% of RE versus 3%) as he included only eyes with >4.0 diopter interocular difference, while we included cases with >1.0 diopter interocular difference which is more appropriate for scientific and statistical purposes.

Jing et al. [15] defined their cutoff values before starting the screening and described test failures of 73% with high cutoff value and 31% with low cutoff value in comparison to 13.4% in general in our study. We defined our cutoff values after obtaining the results which allowed us to locate accurately the smallest VA measure that achieved 100% sensitivity and 96% specificity and defined it as the low cutoff value (at log MAR 0.539, equivalent to 6/18 Snellen's fraction), and a high cutoff value with 100% sensitivity and 89% specificity (at log MAR 0.238, equivalent to 6/10 Snellen's fraction).

Our results were better than Jing et al. [15] even when they tried to improve sensitivity of their screening program by doing simultaneous VA and stereo acuity testing [16]. They had lower sensitivity and specificity than us at their most close low cutoff value to ours in both situations (log MAR of 0.17 = 6/9 Snellen's fraction; 92% sensitivity and 68% specificity with distance VA alone and 94% sensitivity and 66% specificity with combined tests). They still had lower specificity than ours when they used a high cutoff value of 0 log MAR = 6/6 Snellen's fraction, which is rather impractical in screening (100% sensitivity, 22.7% specificity with distance VA alone, and 100% sensitivity, 21% specificity with combined tests). The high sensitivity and specificity of our test were also attributed to the selected older age subjects with better performance of distance VA testing [19, 20].

Very close to our results, Gracia et al., [21] found 80% unilateral amblyopia and 100% anisometropia in their amblyopic cases, in comparison to 100% and 75% in our study, respectively. They used the same criteria of our research of ≥1D interocular difference, while Lee et al. [7], who used an interocular difference of ≥2D, reported amblyopia in 52% of anisometropic eyes. In accordance with other studies on similar age groups, we found neither strabismic nor sensory deprivation amblyopia [22, 23], while we had more amblyopic females than males (*p* = 0.02), in contrary to Chinese population who showed no difference between boys and girls (*P* = 0.782) [15].

Based on our results, we do recommend obligatory regular screening of primary school children using simple distance VA chart by well-trained health care providers. Child referral to consultation is mandatory if having VA of 6/18 or worse in one or both eyes or not adequately corrected by his own glasses. We hope that would help over the lack of awareness among parents and community and promote better quality of life for growing children [8, 24].

## Figures and Tables

**Table 1:** Risk factors affecting amblyopia occurrence.

Risk factors	Amblyopia		*P*
	*n*	Percent	
*Gender*			
Male (193)	1/193	0.5%	0.02^∗^
Female (159)	6/159	3.8%	
*School*			
Public (191)	2/191	1%	0.1
Private (161)	5/161	3.1%	
*Wearing spectacles*			
No (332)	5/332	1.5%	0.008^∗∗^
Yes (20)	2/20	10%	

*n*: number; ^∗^*P* < 0.05 significant; ^∗∗^*P* < 0.01 highly significant.

**Table 2:** Distribution of different RE in all students and in RE cases.

RE type	Number of cases	Percent of each RE type in all (352) students	Percent of each RE type in (47) students with RE
Hyperopia (≥+1.0 diopter)	13	3.6%	27.6%
Myopia (≥−0.5 diopter)	33	9.37%	70.2%
Anisometropia (≥1.0 diopter)	29	8.23%	61.7%
Astigmatism (>1.0 diopter)	12	3.4%	25.5%

**Table 3:** Amblyopia prevalence in different types of RE.

RE type	Amblyopic eyes		Nonamblyopia	
	^∗∗^ *n*	%	*n*	%
Hyperopia (*n* = 13)^∗^	4/13	30.7%	9/13	69.3%
Myopia (*n* = 33)^∗^	3/33	9%	30/33	91%
Anisometropia (*n* = 29)^∗^	7/29	24.1%	22/29	75.8%
Astigmatism (*n* = 12)^∗^	2/12	16.7%	10/12	83.3%

^∗^ *P* > 0.05 not significant; ^∗∗^*n*: number.

**Table 4:** Sensitivity and specificity of VA in the detection of amblyopia (weaker eye inclusion) among all subjects and amblyopic suspected subjects.

Log MAR	Sensitivity	Specificity	PPV	NPV
All subjects ^∗^*n* = 352				
0.238	100%	89%	15.2%	100%
0.389	100%	90%	16.7%	100%
0.539	100%	96%	33.3%	100%
0.690	85%	99%	50%	99.7%
Amblyopia-suspected cases *n* = 47				
0.238	100%	5%	15.6%	100%
0.389	100%	12.5%	16.7%	100%
0.539	100%	65%	33.3%	100%
0.690	85%	85%	50%	97.1%

^∗^ *n*: number.
